# Relationship between Distinct African Cholera Epidemics Revealed via MLVA Haplotyping of 337 *Vibrio cholerae* Isolates

**DOI:** 10.1371/journal.pntd.0003817

**Published:** 2015-06-25

**Authors:** Sandra Moore, Berthe Miwanda, Adodo Yao Sadji, Hélène Thefenne, Fakhri Jeddi, Stanislas Rebaudet, Hilde de Boeck, Bawimodom Bidjada, Jean-Jacques Depina, Didier Bompangue, Aaron Aruna Abedi, Lamine Koivogui, Sakoba Keita, Eric Garnotel, Pierre-Denis Plisnier, Raymond Ruimy, Nicholas Thomson, Jean-Jacques Muyembe, Renaud Piarroux

**Affiliations:** 1 Department of Parasitology and Mycology, Assistance Publique—Hôpitaux de Marseille/Aix-Marseille University, UMR MD3, Marseille, France; 2 Institut National de Recherche Biomédicale, Ministry of Public Health, Kinshasa, Democratic Republic of the Congo; 3 Department of Bacteriology, National Institute of Hygiene, Lomé, Togo; 4 Hôpital d'Instruction des Armées Laveran, Marseille, France; 5 Department of Clinical Sciences, Institute of Tropical Medicine, Antwerp, Belgium; 6 Department of Microbiology, Faculty of Medicine, University of Kinshasa, Kinshasa, Democratic Republic of the Congo; 7 Laboratoire Chrono-Environnement, UMR 6249, CNRS, University of Franche-Comte, Besançon, France; 8 Ministry of Health, Kinshasa, Democratic Republic of the Congo; 9 Institut National de Santé Publique, Conakry, Republic of Guinea; 10 Division Prévention et Lutte contre la Maladie, Ministère de la Santé Publique et de l’Hygiène Publique, Conakry, Republic of Guinea; 11 Royal Museum for Central Africa, Tervuren, Belgium; 12 Clinical Research Department, Nice University Hospital, Nice, France; 13 Pathogen Genomics, Wellcome Trust Sanger Institute, Hinxton, Cambridge, United Kingdom; 14 London School of Hygiene & Tropical Medicine, London, United Kingdom; Beijing Institute of Microbiology and Epidemiology, CHINA

## Abstract

**Background:**

Since cholera appeared in Africa during the 1970s, cases have been reported on the continent every year. In Sub-Saharan Africa, cholera outbreaks primarily cluster at certain hotspots including the African Great Lakes Region and West Africa.

**Methodology/Principal Findings:**

In this study, we applied MLVA (Multi-Locus Variable Number Tandem Repeat Analysis) typing of 337 *Vibrio cholerae* isolates from recent cholera epidemics in the Democratic Republic of the Congo (DRC), Zambia, Guinea and Togo. We aimed to assess the relationship between outbreaks. Applying this method, we identified 89 unique MLVA haplotypes across our isolate collection. MLVA typing revealed the short-term divergence and microevolution of these *Vibrio cholerae* populations to provide insight into the dynamics of cholera outbreaks in each country. Our analyses also revealed strong geographical clustering. Isolates from the African Great Lakes Region (DRC and Zambia) formed a closely related group, while West African isolates (Togo and Guinea) constituted a separate cluster. At a country-level scale our analyses revealed several distinct MLVA groups, most notably DRC 2011/2012, DRC 2009, Zambia 2012 and Guinea 2012. We also found that certain MLVA types collected in the DRC persisted in the country for several years, occasionally giving rise to expansive epidemics. Finally, we found that the six environmental isolates in our panel were unrelated to the epidemic isolates.

**Conclusions/Significance:**

To effectively combat the disease, it is critical to understand the mechanisms of cholera emergence and diffusion in a region-specific manner. Overall, these findings demonstrate the relationship between distinct epidemics in West Africa and the African Great Lakes Region. This study also highlights the importance of monitoring and analyzing *Vibrio cholerae* isolates.

## Author Summary

Cholera is caused by the toxigenic bacterium *Vibrio cholerae*. Since cholera was imported into the West African country of Guinea in 1970, cases have been reported on the continent every year. In Sub-Saharan Africa, cholera occurs in a heterogeneous manner; outbreaks primarily cluster at certain hotspots including the African Great Lakes Region and West Africa. To gain further insight into the mechanisms by which cholera outbreaks emerge and diffuse, we performed genetic analyses of 337 *Vibrio cholera* isolates from the Democratic Republic of the Congo (DRC), Zambia, Guinea and Togo. Isolates from both patients and environmental samples were examined. Our findings demonstrate the relationship between distinct epidemics in West Africa and the African Great Lakes Region. For example, certain strains in the DRC have circulated in the region over a period of several years, occasionally giving rise to expansive epidemics. We also found that the six environmental isolates in our panel were unrelated to the epidemic isolates. Such insight into the country- and region-specific dynamics of the disease is critical to implement optimized public health strategies to control and prevent cholera epidemics. This study also highlights the importance of analyzing *Vibrio cholerae* isolates to complement epidemiological studies.

## Introduction

Since 1817, seven cholera pandemics have plagued humans worldwide [1]. During the current pandemic, the disease first appeared on the African continent when *Vibrio cholerae* was imported by migrants traveling to the Guinean capital of Conakry in 1970 [1]. Following this importation of the bacterium, cholera cases have been reported every year in Africa, and many regions in Sub-Saharan Africa have been deemed cholera endemic [2].

Although Africa currently has the highest incidence of cholera globally, the disease affects the continent in a heterogeneous manner. African cholera outbreaks primarily cluster at certain hotspots including (1) the African Great Lakes Region and (2) West Africa, stretching from Cameroon and the Lake Chad region along the coast to Guinea [3–6]. Cholera outbreaks commencing in the African Great Lakes Region have been found to spread to neighboring countries, such as Sudan in 2006 and Kenya in 2009 [5]. Likewise, epidemics have progressively moved along the West African coast, as observed in 2003–2007 when outbreaks spread from Liberia and Sierra Leone to Guinea [6]. Indeed, cholera appears to spread in a highly dynamic manner that poses a significant public health threat at a regional level across Africa.

To design effective public health strategies to combat the disease, it is critical to understand the mechanisms of cholera emergence and diffusion in a region-specific manner. Epidemiological analysis of outbreaks is critical to identify hotspots and patterns of disease spread. However, molecular biological methods can provide further insight into the relationship between pathogenic strains and epidemic populations [7]. Indeed, isolate typing is useful to differentiate between different isolates, identify clusters, establish phylogeny, and track bacterial transmission. Lam et al. [8] have recently shown that MLVA (Multi-Locus VNTR (Variable Number Tandem Repeat) Analysis) represents a highly discriminatory technique to distinguish between closely related seventh pandemic isolates. They have also emphasized that the method is best applied for outbreak investigations or to identify the source of an outbreak. Our research group has recently demonstrated that MLVA-based analysis of clinical *V*. *cholerae* isolates combined with an epidemiological assessment was instrumental in deciphering the origin of the 2012 Guinean epidemic [9].

In the current study, we applied MLVA-based typing of 337 *V*. *cholerae* isolates from recent cholera epidemics in Sub-Saharan Africa to assess the relationship between outbreaks. We identified 89 unique MLVA haplotypes across our isolate collection. When coupled with corresponding epidemiological data, we revealed the short-term divergence and microevolution of these *V*. *cholerae* populations to provide insight into the dynamics of cholera outbreaks and the relationship between distinct epidemics in West Africa and the African Great Lakes Region.

## Materials and Methods

### *V*. *cholerae* isolates

Overall, we analyzed 337 *V*. *cholerae* isolates derived from epidemics in the Democratic Republic of the Congo (DRC), Guinea, Togo and Zambia. Our panel included six isolates from environmental samples and 331 clinical samples. A total of 237 *V*. *cholerae* isolates from epidemics in the DRC occurring in 2008 (3 isolates), 2009 (108 isolates), 2011 (60 isolates), 2012 (44 isolates) and 2013 (22 isolates) were provided by the INRB (French acronym for the National Institute of Biomedical Research), Kinshasa, DRC. Of the 60 DRC isolates from 2011, two were isolated from environmental water samples collected from Lake Tanganyika by staff at the Centre de recherche en Hydrobiologie (in Uvira, RDC) and analyzed at the Hôpital Générale de Référence in Uvira by Hilde de Boeck. The Guinean reference laboratory of the Public Health National Institute (INSP—Institut National de Santé Publique), with support from the AFRICHOL Consortium (http://www.africhol.org/), provided 36 *V*. *cholerae* isolates collected throughout Guinea during the 2012 epidemic as previously described [9]. The National Institute of Hygiene in Lomé, Togo provided 35 *V*. *cholerae* isolates from Togo, which corresponded to epidemics in 2010 (13 isolates), 2011 (10 isolates) and 2012 (12 isolates provided by the bacteriology laboratory of the National Institute of Hygiene in Togo via the project AFRICHOL). A total of 27 *V*. *cholerae* clinical isolates from the 2012 Zambian epidemic were analyzed; the isolates were collected by the staff at the Cholera Treatment Center at the Mpulungu Health Centre during the CHOLTIC project together with the Institute of Tropical Medicine in Antwerp, Belgium. Two environmental isolates were also collected during the CHOLTIC project in association with the Department of Fisheries, Lake Tanganyika Research Unit. The Mpulungu Health Centre performed the initial characterization of the *V*. *cholerae* environmental samples.

### Ethics statement

Regarding the isolates from Zambia, suspected cholera cases were routinely cultured to test for the presence of *V*. *cholerae*. The study, including a waiver of written consent, was approved by the University of Zambia Biomedical Research Ethics Committee, the Institute of Tropical Medicine institutional review board, and the ethical committee of the University of Antwerp, Belgium (study registration number B300201317249). Patients, children’s parents and/or legal guardians were informed and approved via oral consent before enrollment into the study. Oral consent was registered by the ward nurse, and participant samples received a study ID number to anonymize the data.

Concerning the Guinean isolates, the sampling of suspected cholera cases for culture confirmation of *V*. *cholerae* is included among the routine procedures in accordance with the policies of the Ministry of Health Guinean. The Ministry of Public Health and Public Hygiene, Conakry (Ministre de la Santé Publique et de l’Hygiène Publique) approved the use of these *V*. *cholerae* isolates for research and publication purposes.

In Togo, to confirm suspected cholera cases, patient samples are routinely cultured to test for the presence of *V*. *cholerae*. The directorate of the National Institute of Hygiene, Lomé, Togo (the laboratory director and the head of the bacteriology department) approved the study of these isolates for research purposes, including the comparison of these Togolese isolates with other African *V*. *cholerae* samples.

In the Democratic Republic of the Congo, samples from suspected cholera cases are routinely collected and analyzed for the presence of *V*. *cholerae* in the framework of the epidemiological surveillance program of the Ministry of Health. The ethics committee at the University of Kinshasa approved the analysis of these isolates for research purposes.

All data analyzed in the study were anonymized.

### *V*. *cholerae* culture and DNA extraction

The isolates were subcultured and inoculated into *Vibrio cholerae* Enrichment Broth vials (Bio-Rad). The Bio-Rad vials were subsequently expedited (2–3 days) at ambient temperature to L'Hôpital d'Instruction des Armées Laveran in Marseille, France. In Marseille, the strains were recultivated on non-selective trypticase soy agar medium (Difco Laboratories/BD) for 24 hours at 37°C. Suspected *V*. *cholerae* colonies were identified via Gram-staining, oxidase reaction and agglutination assessment with *V*. *cholerae* O1 polyvalent antisera (Bio-Rad).

For DNA extraction, an aliquot of cultured cells (approximately 50 colonies) was suspended in 500 μL NucliSENS easyMAG lysis buffer (bioMérieux, Marcy l'Etoile, France). Total nucleic acid was extracted from *V*. *cholerae* cultures using a NucliSENS easyMAG platform (bioMérieux) according to the manufacturer’s instructions. Nucleic acid concentration and 260/280 ratio were measured using a NanoDrop 3300 fluorospectrometer (Thermo Scientific, Villebon sur Yvette, France). The supernatants (100 μL) were stored at -20°C for downstream applications.

### MLVA-based typing

Genotyping of the *V*. *cholerae* isolates was performed via MLVA of six VNTRs, including five previously described assays and a novel VNTR assay, VCMS12, specifically designed for this study to improve the discriminative power of the analysis (Table 1) [9–11]. The VCMS12 assay was designed based on the reference strain El Tor N16961 (GenBank accession numbers AE003852.1 and AE003853.1) using Perfect Microsatellite Repeat Finder (currently unavailable). VCMS12 is located within the cholera toxin A subunit promoter region at position 1568189 on chromosome 1 of El Tor N16961. This polymorphic tandem heptanucleotide repeat region has been previously identified by Naha et al [12]. Specific primer pairs were subsequently designed using Primer3 (http://simgene.com/Primer3). The fluorescent-labeled primers (Table 1) were purchased from Applied Biosystems.

**Table 1:** Characteristics and primer sequences of the six tested *V*. *cholerae* VNTRs.

VNTR Locus name	Repeated pattern	Chr.^1^	Position^2^	Primer sequence (5'→3')	Ref.
**VC1**	AACAGA	1	137106	fw: CGGATACTCAAACGCAGGAT	[10,11]
				rv: 6FAM-CTTTCGGTCGGTTTCTCTTG	
**VC4**	TGCTGT	2	187759	fw: TGTTTGAGAGCTCGCCTCTT	[10,11]
				rv: PET-TCATCAAGATGCACGACACA	
**VC5**	GATAATCCA	1	1915539	fw: AGTGGGCACAGAGTGTCAAA	[10,11]
				rv: VIC-AATTGGCCGCTAACTGAGTG	
**VC9**	GACCCTA	1	467111	fw: CGTTAGCATCGAAACTGCTG	[10,11]
				rv: NED-AGAAAACAATCGCCTGCTTG	
**LAV6**	ACCAGA	2	303939	fw: NED-GCCTCCTCAGAAGTTGAGAATC	[9]
				rv: CCGATGAACTCTCTGAACTGG	
**VCMS12**	TTTTGAT	1	1568189	fw: VIC-GAGGTCTAGAATCTGCCCGA	Present study
				rv: AAGCGCTGTGGGTAGAAGTG	

^**1**^ Chr.: chromosome.

^2^ Based on the reference strain El Tor N16961 (GenBank accession numbers: AE003852.1 and AE003853.1).

Each VNTR locus was amplified separately. DNA amplification was carried out by preparing a PCR mix containing the following components: 0.375 μL of each primer (20 μM), 1 X LightCycler 480 Probes Master (Roche Diagnostics) and approximately 100 ng of template DNA. The PCR mix was brought to a total volume of 30 μL with H_2_O. PCR was performed using a LightCycler 480 System (Roche Diagnostics). All PCRs were performed using the thermal cycling conditions as follows: 95°C for 5 min; followed by 30 cycles of 95°C for 30 sec, 58°C for 30 sec and 72°C for 45 sec; 72°C for 5 min.

Aliquots of the PCR products were first diluted 1:30 in sterile water. Next, 1 μL of the diluted PCR reaction was aliquoted into a solution containing 25 μL Hi-Di Formamide 3500 Dx Series (Applied Biosystems) and 0.5 μL GeneScan 500 LIZ Size Standard (Applied Biosystems). The fluorescent end-labeled PCR amplicons were separated via capillary electrophoresis using an ABI PRISM 310 Genetic Analyzer (Applied Biosystems) with POP-7 Polymer (Applied Biosystems). Finally, amplicon size was determined using GeneMapper v.3.0 software (Applied Biosystems).

### Data analysis

The MLVA results were exported to Microsoft Excel 2008 v. 12.2.0. Allele numbers were derived directly from the fragment sizes, and MLVA types were determined from the combined profile of alleles (i.e., each unique combination of six allele numbers was assigned a novel MLVA type number). The absence of allele at a given locus (i.e., no amplification, despite repeated attempts) was assigned “999” for further analyses. No amplification was observed for 22 of the 2022 examined alleles. To perform the Minimum Spanning Tree (MST) analysis, the isolates were further assigned into 10 epidemic populations as follows: DRC 2008, DRC 2009 A (collected in January-May), DRC 2009 B (collected in July-November), DRC 2011, DRC 2012, DRC 2013, Guinea 2012, Zambia 2012, Togo 2010, Togo 2011 and Togo 2012. The DRC 2009 epidemic isolates were sub-grouped by time period as two distinct epidemics were observed in the region during field investigations.

### Cartography

The maps were generated using QGIS version 2.4.0-Chugiak with shapefiles obtained from DIVA-GIS (http://www.diva-gis.org/gdata).

### Minimum Spanning Tree

Based on allelic profiles the evolutionary relationship between all 337 isolates was assessed with the MST algorithm in BioNumerics (Applied Maths, Sint-Martens-Latem, Belgium) using the default settings according to the manufacturer’s recommendations. The MST was constructed using a categorical coefficient as previously described [13].

### goeBURST analysis

To identify clonal complexes and founder MLVA types among the 331 clinical isolates (i.e., excluding the six environmental isolates), MLVA-types were compared at each of the six VNTR loci and genetic relatedness between the strains was assessed using goeBURST version 1.2.1 (http://goeburst.phyloviz.net/) [14]. The goeBURST algorithm identifies mutually exclusive groups of related MLVA types in a population. The algorithm also predicts the presumed founder(s) of each clonal complex and any single locus variant (SLV) and double locus variant (DLV) derivatives. The primary founder of a group is defined as the MLVA type that has the greatest number of SLVs. goeBURST then constructed a spanning forest in which each MLVA type is a node and two MLVA types are connected if they are SLVs. An MLVA cluster was defined as a group of isolates that share identical alleles at five of the six loci with at least one other member of the group. Accordingly, singletons were defined as MLVA types having at least two allelic mismatches with all other MLVA types. The number of re-samplings for bootstrapping was set at 1000.

### Population genetics calculations

Population genetic analyses were performed on each set of epidemic isolates for which there were 10 or more isolates per set; therefore, the three DRC 2008 isolates were excluded from the statistical analyses. Fst (F-statistics; also known as fixation indices) and p-values were calculated pair-wise for all epidemic populations of the 328 clinical isolates using the Nei (1987) method implemented in GenoDive 2.0b25 [15] as were the allelic diversity of all clusters and loci.

### Field investigations

Several field investigations were conducted in the DRC, Guinea and Togo by members of our team (Renaud Piarroux, Stanislas Rebaudet, Berthe Miwanda, Didier Bompangue, Aaron Aruna Abedi, Jean-Jacques Depina and Sandra Moore). Field investigations were performed at sites affected by cholera, in which the data collected included number of cases/deaths, laboratory results, the locales affected and the spatiotemporal evolution of the epidemics. Further details of the field investigations are provided in the corresponding studies of the specific epidemics (concerning the Togo field investigation, please see S1 Text) [9, 16].

In Zambia, strains collected in the context of the national surveillance system and the CHOLTIC project with the objective of comparing strains collected in the vicinity of Lake Tanganyika (at sites of varying distances from the coast). Strains were collected in Mpulungu, Northern Province, between the 12th and 24th of August 2012, during an outbreak that occurred at this time. The following data corresponding to the collected strains was also compiled: patient name, sex, age, date seen at facility, date of symptom onset, laboratory results (if any were performed) and date of patient discharge. No corresponding field investigation was performed during this outbreak.

## Results

### Minimum Spanning Tree and goeBURST analyses of 337 *V*. *cholerae* isolates from West and Central Africa

**Fig 1:**
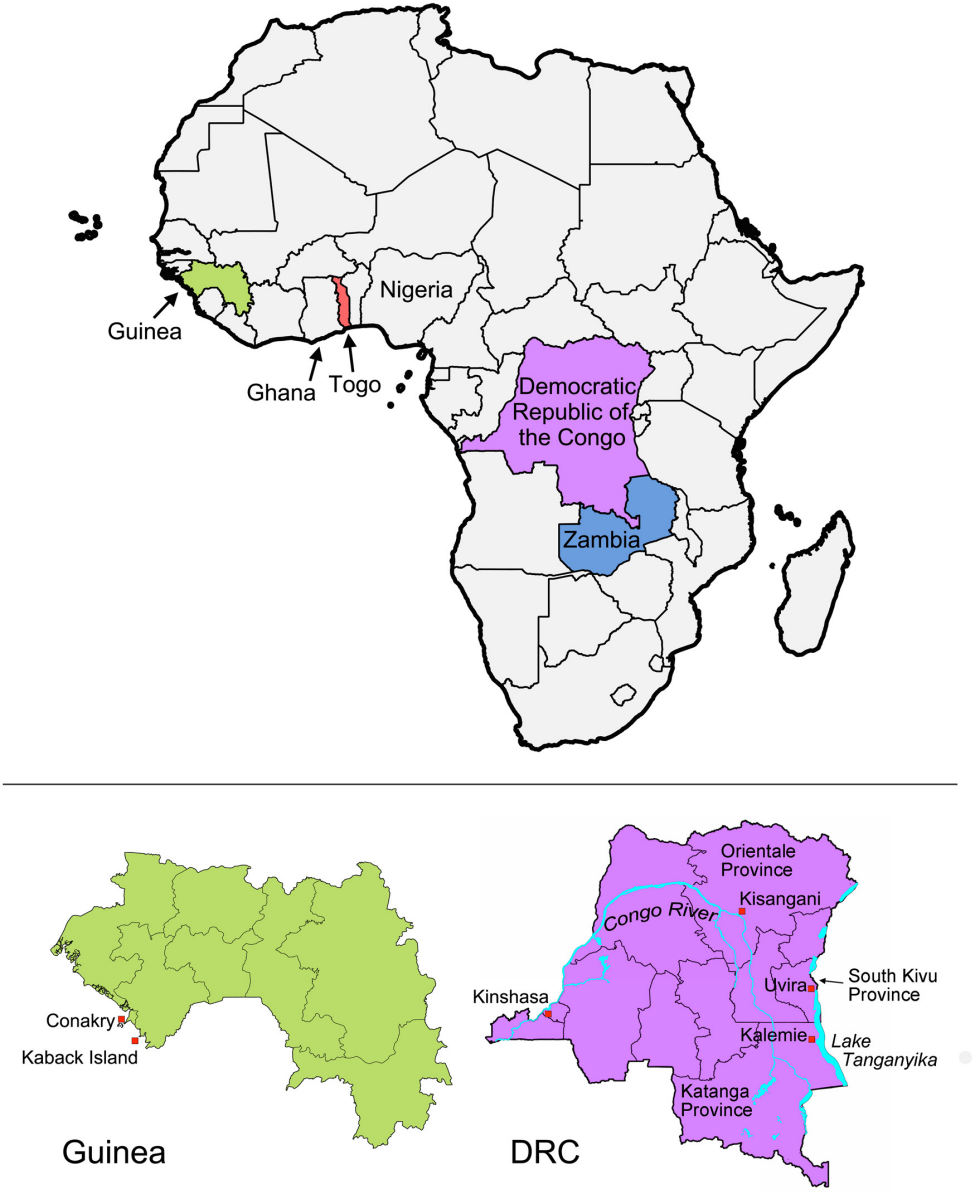
Map of African countries implicated in study. Upper panel: the four countries from which *V*. *cholerae* isolates were collected are indicated in different colors on the map of Africa (i.e., Guinea, green; Togo, red; Democratic Republic of the Congo (DRC), violet and Zambia: blue). Ghana and Nigeria are also labeled. Lower panel: detailed maps of Guinea and the DRC are shown. Lower right: DRC; the cities (red squares) and provinces mentioned are indicated on the map. The Congo River and Lake Tanganyika are also shown (in blue). Lower left: Guinea; the capital, Conakry, and Kaback Island are indicated along the Atlantic coast.

A total of 337 *V*. *cholerae* isolates from recent cholera epidemics in the DRC, Zambia, Guinea and Togo were subjected to MLVA. Each country is localized on the map of Africa in Fig 1. Analysis of the six VNTRs yielded 89 MLVA types. The VNTR loci and epidemic populations (grouped by country and year of isolation) corresponding to each MLVA type are outlined in S1 Table.

**Fig 2:**
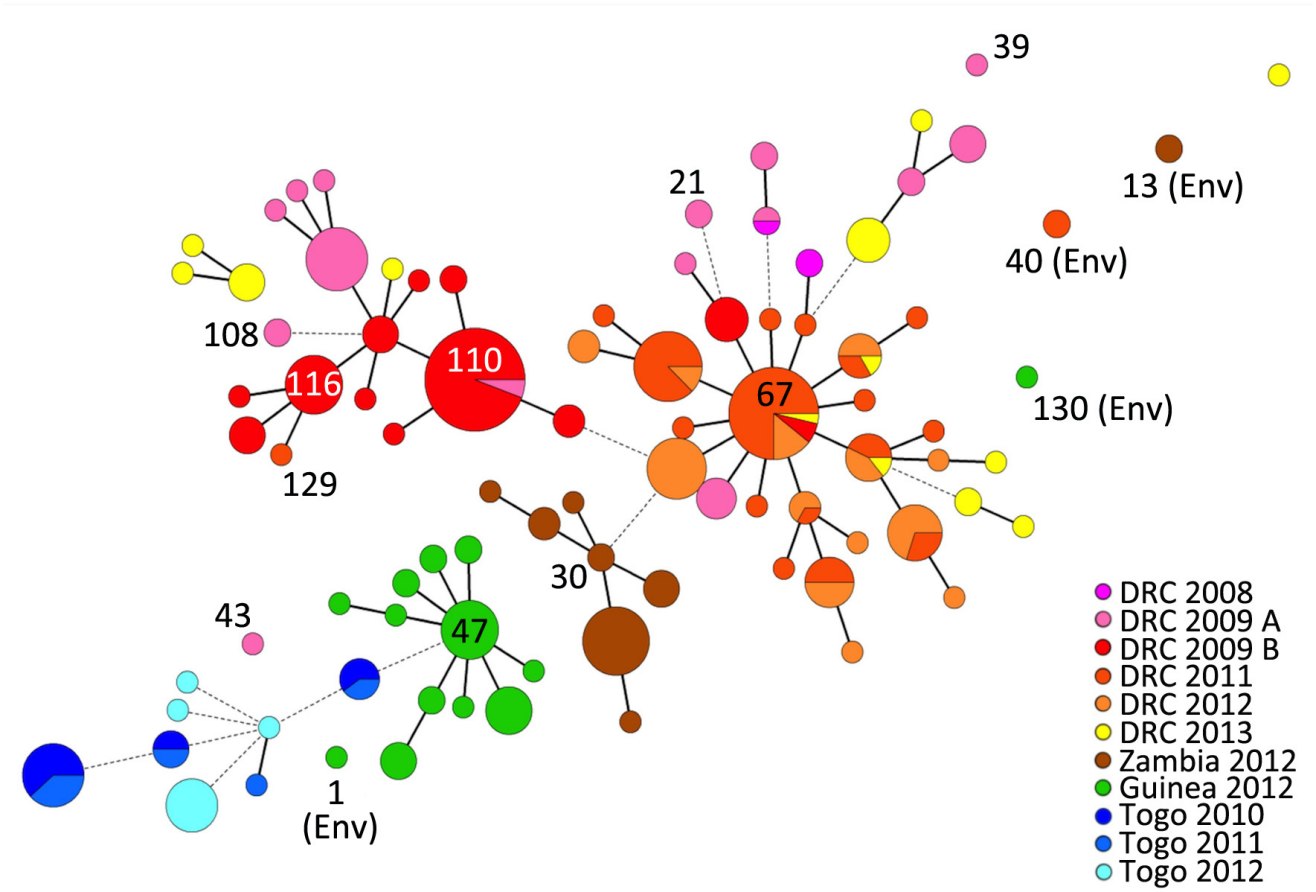
Minimum Spanning Tree based on the MLVA types of 337 *V*. *cholerae* isolates derived from several recent African epidemics. Each MLVA type is represented by a node, and the size of the nodes reflects the number of isolates with the indicated MLVA type. The relationships between isolate MLVA types are indicated by the type of connecting segments and the length of the segment between nodes. The solid lines indicate the most likely SLV, and the dashed lines represent DLV. The distance between the nodes represents the number of varying VNTRs. The colors reflect the distinct country and period of isolate origin (grouped by epidemic populations). Pie charts were used to indicate the distribution of strains isolated from different time periods or countries displaying identical an MLVA type.

A MST was constructed using the combined MLVA data to assess the relationships between the 337 *V*. *cholerae* isolates and the epidemic populations. On a continental scale, the MST revealed strong geographical clustering with isolates from the African Great Lakes Region, including the DRC and Zambia, clustering together. Furthermore, the isolates collected in the West African countries of Guinea and Togo formed a separate group (Fig 2). All but seven clinical isolates from the DRC and Zambia 2012 were linked by one- or two-VNTR variations. Likewise, all clinical isolates from Guinea 2012 and Togo were linked by one- or two-VNTR divergences. At a country-level scale our analyses revealed several distinct clonal groups, most notably (1) DRC 2011/2012, (2) DRC 2009, (3) Zambia 2012 and (4) Guinea 2012. We used goeBURST to identify a potential founder MLVA haplotype for each MLVA cluster. Each epidemic complex was characterized by a central founder MLVA haplotype and closely related derivative SLV or DLV MLVA haplotypes, which branched from the founder. In contrast, all six of the typed environmental isolates were found to be singletons, unrelated to the main clinical epidemic isolate clusters (Fig 2).

Our analysis showed that the DRC 2011 and DRC 2012 isolates grouped together as one discrete complex (Fig 2). During this two-year period in the DRC, cholera was caused by the extensive expansion and diversification from a single MLVA haplotype. The isolate found at the beginning of the 2011/2012 epidemic in Kisangani, Orientale Province in March 2011 was haplotype #67, which was designated the founder of the DRC 2011/2012 complex. MLVA type #67 and a SLV of this haplotype were the only types identified during the first week of the outbreak. This MLVA cluster then diversified in parallel with the spatiotemporal spread of the epidemic [16], as the most distant MLVA haplotypes within this cluster were identified in distant provinces in 2012. These findings correlate with an epidemiological report of the cholera epidemic that struck the DRC in 2011. This epidemic aggressively diffused from the onset point in Kisangani, Orientale Province across the country in less than 130 days. Strikingly, outbreaks followed the Congo River and quickly reached non-endemic zones in the West that had not experienced an epidemic for approximately 10 years [16]. Kisangani and the Congo River are localized on the detailed map of the DRC (Fig 1, lower right).

Interestingly, the predicted founder of the 2011/2012 DRC epidemic, persisted in the country over the course of several years, as haplotype #67 was represented in isolates collected in the DRC during the 2009, 2011, 2012 and 2013 epidemics. Only one DRC isolate (MLVA type #129) collected in 2011 did not belong to the major DRC 2011/2012 MLVA cluster. Instead, haplotype #129 was a SLV of the DRC 2009 haplotype #116 cluster. In stark contrast, the *V*. *cholerae* non-O1 strain isolated at the same period from a water sample in Uvira, South Kivu was a genetically unrelated singleton (MLVA type #40) (Fig 2). Uvira is located on the northern shores of Lake Tanganyika as indicated in Fig 1 (lower right panel).

Overall, the panel of DRC 2009 isolates displayed a high level of genetic diversity. In fact, four DRC 2009 clinical isolates (MLVA types #39, #43, #21 and #108) collected in February and March 2009 were designated distantly related singletons (Fig 2). The MST was then analyzed in further detail considering the date of sample isolation. The isolates collected during the first half of the year were highly diverse (indicated as “DRC 2009 A” in pink; Fig 2). In contrast, 63 of 66 isolates (95.5%) collected from July to November of 2009 in Katanga Province formed a tight clonal complex (indicated as “DRC 2009 B” in red; Fig 2). This bottleneck effect was concomitant with an epidemic rebound in Katanga Province after a complete lull in cholera transmission in May and June 2009. In July 2009, cholera first appeared in Kalemie, a city located on the shore of Lake Tanganyika, and then spread throughout the rest of the province (based on field investigations in the DRC; Renaud Piarroux). MLVA types #110 and #116 were designated potential founders of this DRC 2009 B MLVA complex. Interestingly, MLVA type #110 was the first isolate collected on February 5, 2009, and isolates harboring this haplotype were collected up to November 20, 2009. Notably, MLVA type #110 was also found in August 2009 in Uvira, a city located approximately 360 km north of Kalemie on the shore of Lake Tanganyika (all sites are labeled in the lower right panel of Fig 1). Therefore, we hypothesize that this strain likely persisted in the region following the early-2009 outbreaks and a subsequently gave rise to the late-2009 DRC epidemic.

In Zambia, all clinical isolates from the 2012 epidemic formed a restricted clonal complex, which derived from the predicted founder MLVA #30. Once more, the two non-O1 environmental isolates collected from the shores of Lake Tanganyika in Mpulungu, Northern Province were singletons (MLVA haplotype #13) unrelated to the clonal complex (Fig 2).

The Guinea 2012 clinical isolates formed a solid clonal complex, with an MST of closely related derivative isolates that stemmed from the founder haplotype (MLVA type #47) (Fig 2). In a previous study, our group has shown that the Guinea 2012 epidemic appears to be due to the importation of a toxigenic clone from Sierra Leone. Using MLVA typing, we have demonstrated progressive genetic diversification of the strains from the founder type correlated with spatiotemporal epidemic spread [9]. The founding MLVA type was also the first and only MLVA type indentified during the onset of the epidemic, on Kaback Island (Fig 1, lower left), Guinea in February 2012 [9]. In contrast, the two Guinean environmental strains (MLVA types #1 and #130) isolated from water samples at the site of the initial outbreak (Kaback) were unrelated singletons (Fig 2).

Our data showed that the Togo isolates represent a diverse set of MLVA haplotypes, as they were only related to the Guinean isolate MLVA types by a single DLV (Fig 2). Most of the isolates collected in Togo were designated singletons.

### Population genetics

To provide statistical power to the observed relationships between 328 clinical isolates, population genetic analyses were performed. The six unrelated environmental isolates and the three isolates from DRC in 2008 were excluded from this analysis. Taking each MLVA VNTR locus in turn, this analysis revealed that there were 5, 17, 6, 6, 17 and 5 alleles, for the VNTR loci denoted VC1, VC4, VC5, VC9, LAV6 and VC12, respectively (Table 2). The discriminatory power of the six tested VNTRs was calculated via an index of genetic diversity (IOD) analysis (Nei, 1987) using GenoDive 2.0b25. The IOD and PCR product size range for each VNTR is outlined in Table 2. Accordingly, the corrected indices of diversity per locus were 0.758, 0.927, 0.54, 0.512, 0.871 and 0.579 for VC1, VC4, VC5, VC9, LAV6 and VCMS12, respectively. The overall corrected IOD for the six VNTRs combined was 0.698. The two most variable VNTRs were located on the small chromosome, which correlates with the observations reported by Lam et al. [8] (Table 2).

**Table 2:** Indices of genetic diversity per locus and observed PCR product size range.

Locus	Chromosome	Num	Eff num	Pop div	IOD	Corr IOD	PCR product size range (base pairs)
**VC1**	1	5	1.516	0.357	0.717	0.758	179–203
**VC4**	2	17	2.302	0.592	0.893	0.927	169–284
**VC5**	1	6	1.157	0.142	0.5	0.54	160–215
**VC9**	1	6	1.226	0.193	0.48	0.512	160–202
**LAV6**	2	17	2.052	0.537	0.838	0.871	0, 209–329
**VC12**	1	5	1.053	0.053	0.526	0.579	0, 258–286
**Overall**		9.333	1.551	0.312	0.659	0.698	-

Genetic diversity (Nei, 1987) of 328 *V*. *cholerae* isolates, considering six loci, was calculated in GenoDive 2.0b25. Num = number of alleles; Eff num = effective number of alleles; Pop div = gene diversity within populations; IOD = Index of Diversity; Corr IOD = corrected Index of Diversity.

The IOD (based on Nei, 1987) per population was calculated to determine the extent of genetic diversity of each designated population. The epidemics with the highest degree of genetic diversity were DRC 2009 A (IOD = 0.545) and DRC 2013 (IOD = 0.586). In contrast, the epidemic isolates derived from Togo 2012 (IOD = 0.152) displayed the lowest diversity. Epidemics in DRC 2011 (IOD = 0.218), DRC 2012 (IOD = 0.242), Zambia 2012 (IOD = 0.206) and Guinea 2012 (IOD = 0.222) also showed relatively low gene diversity (Table 3).

**Table 3:** Indices of genetic diversity per population.

Population	Number of samples	Number of alleles	Effective number of alleles	Diversity within populations
DRC 2011	58	3.333	1.398	0.218
DRC 2012	44	2.333	1.723	0.242
DRC 2009 A	39	5.5	2.698	0.545
DRC 2009 B	69	3	1.476	0.256
Guinea 2012	34	2.5	1.397	0.222
DRC 2013	22	4.667	2.962	0.586
Togo 2010	13	2	1.582	0.325
Togo 2011	10	2.167	1.764	0.374
Togo 2012	12	2	1.238	0.152
Zambia 2012	27	2	1.313	0.206

Genetic diversity (Nei, 1987) per population of the 328 *V*. *cholerae* isolates was calculated in GenoDive 2.0b25.

Pairwise differentiation analysis was performed to understand the statistical relationship between the epidemic populations. The Fst values for all pairs of populations were calculated considering all 328 *V*. *cholerae* clinical isolates. All Fst and p-values are outlined in Table 4. The closest statistically significant relationship was between the epidemics of DRC 2011 and DRC 2012 (Fst = 0.125, p = 0.001), which is coherent with the rapid diversification and expansion of an epidemic clone [16].

**Table 4:** Pairwise differentiation.

Epidemic population	DRC 2011	DRC 2012	DRC 2009 A	DRC 2009 B	Guinea 2012	DRC 2013	Togo 2010	Togo 2011	Togo 2012	Zambia 2012
**DRC 2011**	--	0.125	0.371	0.607	0.743	0.37	0.706	0.692	0.759	0.637
**DRC 2012**	0.001	--	0.319	0.571	0.732	0.33	0.707	0.69	0.737	0.592
**DRC 2009 A**	0.001	0.001	--	0.276	0.464	0.107	0.444	0.423	0.53	0.429
**DRC 2009 B**	0.001	0.001	0.001	--	0.66	0.353	0.655	0.643	0.714	0.661
**Guinea 2012**	0.001	0.001	0.001	0.001	--	0.516	0.486	0.475	0.693	0.662
**DRC 2013**	0.001	0.001	0.001	0.001	0.001	--	0.447	0.418	0.551	0.476
**Togo 2010**	0.001	0.001	0.001	0.001	0.001	0.001	--	-0.076	0.525	0.658
**Togo 2011**	0.001	0.001	0.001	0.001	0.001	0.001	0.829	--	0.461	--.65
**Togo 2012**	0.001	0.001	0.001	0.001	0.001	0.001	0.001	0.001	--	0.773
**Zambia 2012**	0.001	0.001	0.001	0.001	0.001	0.001	0.001	0.001	0.001	--

The Fst values for all pairs of populations were calculated with GenoDive 2.0b25, considering 328 clinical *V*. *cholerae* isolates and six loci. The Fst values are listed on the top triangle, while the corresponding p-values are listed in the lower triangle.

The DRC 2013 epidemic was closest related to the early DRC 2009 isolates (Fst = 0.107, p = 0.001), which suggests that the 2013 epidemic was likely due to an expansion of strains circulating in the country during pervious epidemics. The pairwise analysis also demonstrated a statistically signification relationship between the early and late 2009 isolates from the DRC (2009 A and 2009 B, respectively) (Fst = 0.276, p = 0.001). These observations further support the hypothesis that the late 2009 epidemic came about following a bottleneck of the early 2009 DRC epidemic isolates.

## Discussion

Overall, our results provide novel insight into the epidemic phenomena of cholera in West and Central Africa. At the sub-regional level, MST analysis revealed two distinct African clusters: (1) the African Great Lakes Region group, comprising DRC and Zambia isolates, and (2) the West African cluster with isolates from Togo and Guinea. At the country level, the epidemic *V*. *cholerae* populations from DRC 2011/2012, Zambia 2012, Guinea 2012 and late-2009 DRC were each designated tight complexes. The expansion of the isolate populations coincided with the progression of each epidemic, as the founder MLVA types corresponded to isolates collected at outbreak onset and more distantly related derivatives represented isolates found later during the epidemic.

Analysis of isolates from the DRC revealed that certain strains appear to remain in circulation in the country over a period of several years and eventually engender explosive outbreaks with diversification of founding isolates, as observed in 2011. This phenomenon is distinct from that observed in Togo, where isolates were grouped into a loosely connected quasi-complex without a founder MLVA type. The Togo isolate results rather indicate that when outbreaks occur, the isolates fail to diversify or diffuse throughout the country. A field assessment of outbreaks in Togo has revealed that the country is vulnerable to importation of cholera cases from neighboring countries, although outbreaks are then quickly extinguished (UNICEF field investigation (Sandra Moore) and personal communication with Dr Adodo Yao Sadji). From 2010 to week 48 of 2014, the country only recorded 551 suspected cholera cases [17–21]. We hypothesize that the Togo isolates rather represent the descendants of a much larger epidemic cluster from a neighboring country experiencing severe cholera epidemics, such as the nearby countries of Ghana and Nigeria. From 2010 to week 48 of 2014, Ghana reported 48,546 suspected cholera cases [17–21]. During that same five-year period, Nigeria signaled a staggering 110,904 suspected cases [17–21]. Isolates from these affected countries would have to be analyzed to test this hypothesis.

As *V*. *cholerae* is autochthonous in the coastal aquatic ecosystem, it has been widely presumed that cholera epidemics are triggered by environmental factors that promote growth of local bacterial reservoirs [22]. However, all six of the environmental isolates collected from a range of countries were genetically unrelated singletons. We acknowledge that additional environmental isolates of *V*. *cholerae* should be included in the panel to affirm the relationship (or lack of) between clinical outbreak strains and those found in water bodies. Indeed, to examine the diversity of environmental strains, efforts should be made to collect further samples. Nevertheless, our preliminary analysis of the environmental samples correlates with two recent reviews elucidating the environmental determinants of cholera outbreaks in Africa. These reviews by Rebaudet et al. [5,6] found that at least 76% of cholera cases in Sub-Saharan Africa occurred in non-coastal regions located over 100 km from the coast. From 2009 to 2011, annual incidence rates of cholera were three times higher in inland Africa compared with the coastal region. In fact, toxigenic *V*. *cholerae* isolates have only been recovered from the environment during an outbreak, when patient-derived contamination of water sources is expected [5,6].

Our findings are also consistent with the phylogenic assessment of an extensive panel of seventh pandemic *V*. *cholerae* isolates. Mutreja et al. [23] have revealed that a specific *V*. *cholerae* El Tor clonal lineage appears to be responsible for the current pandemic. Their study demonstrated that the seventh pandemic is monophyletic and originated from a single ancestral clone that has radiated globally in distinct waves [23]. Lineages of the current pandemic appear to emerge, diversify and eventually become extinct [23]. Notably, we observed a similar phenomenon at a smaller scale with the DRC 2009, DRC 2011/2012, Guinea 2012 and Zambia 2012 epidemics. As isolates from the African Great Lakes Region were not included in the seventh pandemic phylogeny, whole-genome sequencing and phylogenic analysis of this panel of African strains would provide even further insight into the mechanisms of cholera epidemics in the region. Together, MLVA and whole-genome sequencing-based phylogeny represent complementary approaches to better understand epidemic dynamics. MLVA is useful to elucidate the short-term microevolution of clonal complexes, while sequence-based phylogeny enables the identification of distant ancestors and related strains at a global level.

Concerning the limitations of the study, our findings would be bolstered by increased isolate sampling of several years in these and neighboring affected countries. Indeed, we could not verify that MLVA type #67 isolates found in the DRC persisted in the country throughout the 2010 epidemic, as few isolates were collected in 2010 due to a lack of funding for the epidemiological surveillance and prevention of cholera. Likewise, the analysis of the Togolese epidemics would benefit from additional isolate sampling in neighboring countries. Finally, although environmental *V*. *cholerae* samples are difficult to isolate, this study would be enhanced by including additional isolates found in water bodies located in cholera-endemic areas.

Further studies should address the detailed mechanisms of cholera in the zones where cholera appears to persist. If the cholera dilemma in Africa can be narrowed down to a few locales, secondary affected areas (such as perhaps Togo and Guinea) may be largely protected by targeted interventions in cholera epicenters such as the DRC, Ghana and Nigeria. Therefore, with a clear understanding of cholera dynamics in the region, public health resources would be most effectively and efficiently applied.

Overall, our results show that cholera is indeed a regional public health dilemma in Africa. With varying dynamics in each country, certain strains are able to persist in a given region over a period of several years and occasionally spread into non-endemic areas or neighboring countries. Indeed, several elements play a role in cholera epidemics including climate, geography, economy, hygiene, sanitation, access to potable water and population movement, as addressed in the corresponding epidemiological reports (concerning the Togo field investigation, please see S1 Text) [9, 16]. Therefore, public health strategies should be optimized according to the dynamics and scale of cholera epidemics in each region. These findings also demonstrate the importance of monitoring the circulation of the bacterium among human populations, which appear to serve as the principal reservoir of toxigenic *V*. *cholerae*. Combined with classical epidemiological investigations, MLVA represents a rapid and discriminatory tool to track outbreak evolution at an epidemic and regional level.

## Supporting Information

S1 Table The epidemic populations and PCR amplicon size of each allele corresponding to each MLVA type. The number of isolates corresponding to each MLVA type is indicated on the right. The environmental isolates are indicated with an asterisk.
(DOCX)

S1 Text Observations of the field investigation performed in Togo 2014. (DOCX)

## References

[1] Echenberg M. Africa in the Time of Cholera: A History of Pandemics from 1817 to the Present. New York, NY, USA: Cambridge University Press; 2011.

[2] Gaffga NH, Tauxe R V, Mintz ED. Cholera: A New Homeland in Africa? Am Soc Trop Med Hyg. 2007;77(4):705–13. 17978075

[3] Nkoko DB, Giraudoux P, Plisnier P, Tinda AM, Piarroux M. Dynamics of Cholera Outbreaks in Great Lakes Region of Africa, 1978–2008. 2011;17(10 2010):2026–34. 10.3201/eid1711.110170.Dynamics 22099090 PMC3310557

[4] Oger P, Sudre B. Water, sanitation and hygiene and Cholera Epidemiology: An Integrated Evaluation in the Countries of the Lake Chad Basin. UNICEF WCARO. 2011 p. 107. Available from: http://ochaonline.un.org/CoordinationIASC/WASH/tabid/5629/language/fr-FR/Default.aspx

[5] Rebaudet S, Sudre B, Faucher B, Piarroux R. Environmental Determinants of Cholera Outbreaks in Inland Africa: A Systematic Review of Main Transmission Foci and Propagation Routes. J Infect Dis. 2013;208(Suppl 1):S46–S54. 10.1093/infdis/jit195 24101645

[6] Rebaudet S, Sudre B, Faucher B, Piarroux R. Cholera in coastal Africa: a systematic review of its heterogeneous environmental determinants. J Infect Dis. 2013 11 [cited 2013 Nov 14];208 Suppl:S98–S106. Available from: http://www.ncbi.nlm.nih.gov/pubmed/24101653 10.1093/infdis/jit202 24101653

[7] Ramisse V, Houssu P, Hernandez E, Denoeud F, Hilaire V, Lisanti O, et al Variable Number of Tandem Repeats in Salmonella enterica subsp. enterica for Typing Purposes. J Clin Microbiol. 2004;42(12):5722–30. 15583305 10.1128/JCM.42.12.5722-5730.2004 PMC535243

[8] Lam C, Octavia S, Reeves PR, Lan R. Multi-locus variable number tandem repeat analysis of 7th pandemic Vibrio cholerae. BMC Microbiol. BMC Microbiology; 2012;12(82):1–11. 10.1186/1471-2180-12-82 22624829 PMC3438101

[9] Rebaudet S, Mengel MA, Koivogui L, Moore S, Mutreja A, Kande Y, et al Deciphering the Origin of the 2012 Cholera Epidemic in Guinea by Integrating Epidemiological and Molecular Analyses. PLoS Negl Trop Dis. 2014;8(6):e2898 10.1371/journal.pntd.0002898 24901522 PMC4046952

[10] Olsen JS, Aarskaug T, Skogan G, Fykse ME, Ellingsen AB, Blatny JM. Evaluation of a highly discriminating multiplex multi-locus variable-number of tandem-repeats (MLVA) analysis for Vibrio cholerae. J Microbiol Methods [Internet]. Elsevier B.V.; 2009;78(3):271–85. Available from: 10.1016/j.mimet.2009.06.011 19555725

[11] Kendall EA, Chowdhury F, Begum Y, Khan AI, Li S, Thierer JH, et al Relatedness of Vibrio cholerae O1 / O139 Isolates from Patients and Their Household Contacts, Determined by Multilocus Variable-Number Tandem-Repeat Analysis. J Bacteriol. 2010;192(17):4367–76. 10.1128/JB.00698-10 20585059 PMC2937383

[12] Naha A, Chowdhury G, Ghosh-Banerjee J, Senoh M, Takahashi T, Ley B, et al Molecular Characterization of High-Level-Cholera-Toxin-Producing El Tor Variant Vibrio cholerae Strains in the Zanzibar Archipelago of Tanzania. J Clin Microbiol. 2013;51(3):1040–5. 10.1128/JCM.03162-12 23325815 PMC3592071

[13] Choi SY, Lee JH, Jeon YS, Lee HR, Kim EJ, Ansaruzzaman M, et al Multilocus variable-number tandem repeat analysis of Vibrio cholerae O1 El Tor strains harbouring classical toxin B. J Med Microbiol. 2010 7;59(Pt 7):763–9. 10.1099/jmm.0.017939-0 Epub 2010 Mar 18. 20299504

[14] Francisco A, Vaz C, Monteiro P, Melo-Cristino J, Ramirez M, Carriço J. PHYLOViZ: Phylogenetic inference and data visualization for sequence based typing methods. BMC Bioinformatics. 2012;13(87):1–10. 10.1186/1471-2105-13-87 22568821 PMC3403920

[15] Meirmans PG, Van Tienderen PH. GENOTYPE and GENODIVE: two programs for the analysis of genetic diversity of asexual organisms. Mol Ecol Notes. 2004;4:792–4.

[16] Bompangue D, Vesenbeckh SM, Giraudoux P, Castro M, Muyembe J, Ilunga BK, et al Cholera ante portas—The re-emergence of cholera in Kinshasa after a ten-year hiatus. PLOS Curr Disasters. 2012;1:1–12. 10.1371/currents.RRN1310 PMC3299488 22453903

[17] WHO, UNICEF. Cholera outbreak in the West and Central Africa: Regional Update, 2014 (WEEK 49). 2014. Available from: http://www.unicef.org/cholera/files/Cholera_regional_update_W49_2014_West_and_Central_Africa.pdf

[18] Centers for Disease Control and Prevention. Weekly epidemiological record. Cholera. 2014;(31):345–56.

[19] Centers for Disease Control and Prevention. Weekly epidemiological record. Cholera. 2013;(31):321–36. 23980290

[20] Centers for Disease Control and Prevention. Weekly epidemiological record. Cholera. 2012;(31):289–304.

[21] Centers for Disease Control and Prevention. Weekly epidemiological record. Cholera. 2011;(31):325–40. 21800468

[22] Colwell RR. Global Climate and Infectious Disease: The Cholera Paradigm. Science. 1996;274(5295):2025–31. 8953025 10.1126/science.274.5295.2025

[23] Mutreja A, Kim DW, Thomson NR, Connor TR, Lee JH, Kariuki S, et al Evidence for several waves of global transmission in the seventh cholera pandemic. Nature. Nature Publishing Group, a division of Macmillan Publishers Limited. All Rights Reserved.; 2011 9 22;477(7365):462–5. Available from: 10.1038/nature10392 PMC3736323 21866102

