# Supplementary material for: Relationship between Distinct African Cholera Epidemics Revealed via MLVA Haplotyping of 337 Vibrio cholerae Isolates
Source: PLoS Negl Trop Dis. 2015 Jun 25;9(6):e0003817. doi: 10.1371/journal.pntd.0003817 (PMC4482140; doi:10.1371/journal.pntd.0003817)
Supplement: S1 Table — The number of isolates corresponding to each MLVA type is indicated on the right. The environmental isolates are indicated with an asterisk. (DOCX) [file pntd.0003817.s001.docx]

**S1 Table. The epidemic populations and PCR amplicon size of each allele corresponding to each MLVA type.**

| **Epidemic** | **MLVA type** | **Locus** | | | | | | **Num of isolates** |
| --- | --- | --- | --- | --- | --- | --- | --- | --- |
|  |  | **VC1** | **VC4** | **VC5** | **VC9** | **LAV6** | **VCMS12** |  |
| **Guinea 2012** | 1* | 0 | 0 | 185 | 188 | 281 | 0 | 1 |
| **Zambia 2012** | 2 | 179 | 203 | 197 | 188 | 245 | 272 | 15 |
| **Zambia 2012** | 3 | 179 | 203 | 215 | 188 | 245 | 272 | 1 |
| **DRC 2013** | 4 | 179 | 226 | 178 | 160 | 0 | 0 | 1 |
| **Togo 2010, 2011** | 5 | 179 | 243 | 188 | 188 | 251 | 272 | 5 |
| **Zambia 2012** | 13* | 185 | 192 | 157 | 160 | 0 | 0 | 2 |
| **DRC 2009 A** | 21 | 185 | 198 | 197 | 181 | 233 | 265 | 2 |
| **DRC 2013** | 22 | 185 | 198 | 197 | 188 | 257 | 265 | 6 |
| **DRC 2013** | 23 | 185 | 198 | 197 | 188 | 263 | 265 | 1 |
| **DRC 2009 A** | 25 | 185 | 198 | 197 | 188 | 287 | 265 | 2 |
| **DRC 2009 A** | 26 | 185 | 198 | 197 | 188 | 293 | 265 | 4 |
| **DRC 2011** | 29 | 185 | 203 | 197 | 188 | 233 | 265 | 1 |
| **Zambia 2012** | 30 | 185 | 203 | 197 | 188 | 245 | 272 | 2 |
| **Zambia 2012** | 31 | 185 | 203 | 197 | 188 | 251 | 0 | 1 |
| **Zambia 2012** | 32 | 185 | 203 | 197 | 188 | 251 | 272 | 3 |
| **Zambia 2012** | 33 | 185 | 203 | 197 | 188 | 263 | 272 | 1 |
| **DRC 2008, 2009 A** | 35 | 185 | 203 | 197 | 195 | 287 | 265 | 2 |
| **Zambia 2012** | 36 | 185 | 209 | 197 | 188 | 245 | 272 | 4 |
| **DRC 2009 A** | 37 | 185 | 209 | 197 | 195 | 287 | 265 | 2 |
| **DRC 2011** | 38 | 185 | 215 | 197 | 188 | 233 | 265 | 1 |
| **DRC 2009 A** | 39 | 185 | 215 | 197 | 202 | 287 | 258 | 1 |
| **DRC 2011** | 40* | 185 | 215 | 203 | 195 | 0 | 0 | 2 |
| **Togo 2010, 2011** | 41 | 185 | 220 | 188 | 160 | 233 | 272 | 13 |
| **DRC 2013** | 42 | 185 | 220 | 197 | 188 | 239 | 265 | 1 |
| **DRC 2009 A** | 43 | 185 | 232 | 160 | 167 | 329 | 286 | 1 |
| **Guinea 2012** | 44 | 185 | 243 | 188 | 181 | 287 | 272 | 4 |
| **Guinea 2012** | 45 | 185 | 243 | 188 | 188 | 269 | 272 | 2 |
| **Guinea 2012** | 46 | 185 | 243 | 188 | 188 | 275 | 272 | 1 |
| **Guinea 2012** | 47 | 185 | 243 | 188 | 188 | 281 | 272 | 11 |
| **Guinea 2012** | 48 | 185 | 243 | 188 | 188 | 287 | 272 | 2 |
| **Guinea 2012** | 49 | 185 | 243 | 188 | 188 | 311 | 272 | 1 |
| **Guinea 2012** | 50 | 185 | 243 | 188 | 195 | 281 | 272 | 2 |
| **Guinea 2012** | 51 | 185 | 249 | 188 | 188 | 281 | 272 | 2 |
| **Guinea 2012** | 52 | 185 | 255 | 188 | 188 | 275 | 272 | 1 |
| **Guinea 2012** | 53 | 185 | 255 | 188 | 188 | 281 | 272 | 7 |
| **Guinea 2012** | 54 | 185 | 272 | 188 | 188 | 281 | 272 | 1 |
| **DRC 2009 B** | 62 | 191 | 198 | 197 | 188 | 233 | 265 | 6 |
| **DRC 2011** | 63 | 191 | 198 | 197 | 188 | 239 | 265 | 1 |
| **DRC 2012, 2011** | 65 | 191 | 203 | 197 | 188 | 227 | 265 | 3 |
| **DRC 2011** | 66 | 191 | 203 | 197 | 188 | 233 | 0 | 1 |
| **DRC 2011, 2012, 2009 B, 2013** | 67 | 191 | 203 | 197 | 188 | 233 | 265 | 28 |
| **DRC 2011, 2012, 2013** | 68 | 191 | 203 | 197 | 188 | 239 | 265 | 7 |
| **DRC 2012** | 69 | 191 | 203 | 197 | 188 | 245 | 265 | 12 |
| **DRC 2011** | 70 | 191 | 203 | 197 | 188 | 257 | 265 | 1 |
| **DRC 2009 A** | 71 | 191 | 203 | 197 | 188 | 281 | 265 | 5 |
| **DRC 2011** | 72 | 191 | 203 | 206 | 188 | 233 | 265 | 1 |
| **DRC 2012** | 73 | 191 | 209 | 197 | 181 | 227 | 265 | 1 |
| **DRC 2011, 2012** | 74 | 191 | 209 | 197 | 188 | 227 | 265 | 8 |
| **DRC 2011** | 75 | 191 | 209 | 197 | 188 | 233 | 0 | 1 |
| **DRC 2011, 2012** | 76 | 191 | 209 | 197 | 188 | 233 | 265 | 16 |
| **DRC 2011, 2012** | 77 | 191 | 209 | 197 | 188 | 239 | 265 | 10 |
| **DRC 2012** | 78 | 191 | 209 | 197 | 188 | 245 | 265 | 3 |
| **DRC 2012** | 80 | 191 | 209 | 197 | 195 | 239 | 265 | 1 |
| **DRC 2012** | 81 | 191 | 215 | 197 | 188 | 227 | 265 | 1 |
| **DRC 2011, 2012, 2013** | 82 | 191 | 215 | 197 | 188 | 233 | 265 | 6 |
| **DRC 2012** | 83 | 191 | 220 | 197 | 188 | 239 | 265 | 1 |
| **Togo 2010, 2011** | 84 | 191 | 232 | 188 | 160 | 233 | 272 | 4 |
| **DRC 2008** | 85 | 191 | 232 | 197 | 188 | 257 | 265 | 2 |
| **Togo 2012** | 88 | 191 | 238 | 188 | 160 | 275 | 272 | 9 |
| **Togo 2012** | 89 | 191 | 243 | 188 | 160 | 251 | 272 | 1 |
| **Togo 2011** | 90 | 191 | 243 | 188 | 160 | 257 | 272 | 1 |
| **DRC 2009 B** | 96 | 191 | 260 | 188 | 188 | 245 | 265 | 3 |
| **Togo 2012** | 97 | 191 | 266 | 188 | 160 | 239 | 272 | 1 |
| **Togo 2012** | 98 | 191 | 272 | 188 | 160 | 245 | 272 | 1 |
| **DRC 2013** | 99 | 197 | 169 | 188 | 181 | 221 | 265 | 1 |
| **DRC 2009 A** | 100 | 197 | 198 | 197 | 188 | 233 | 265 | 1 |
| **DRC 2011** | 101 | 197 | 203 | 197 | 188 | 227 | 265 | 1 |
| **DRC 2011** | 102 | 197 | 203 | 197 | 188 | 233 | 265 | 1 |
| **DRC 2013** | 105 | 197 | 215 | 197 | 188 | 239 | 265 | 2 |
| **DRC 2013** | 106 | 197 | 215 | 197 | 188 | 245 | 265 | 1 |
| **DRC 2009 A** | 108 | 197 | 238 | 188 | 188 | 209 | 265 | 2 |
| **DRC 2009 B** | 109 | 197 | 260 | 188 | 188 | 245 | 0 | 1 |
| **DRC 2009 A & B** | 110 | 197 | 260 | 188 | 188 | 245 | 265 | 35 |
| **DRC 2009 B** | 111 | 197 | 260 | 188 | 188 | 257 | 265 | 2 |
| **DRC 2013** | 112 | 197 | 266 | 188 | 188 | 227 | 265 | 1 |
| **DRC 2009 B** | 113 | 197 | 266 | 188 | 188 | 239 | 265 | 1 |
| **DRC 2009 B** | 114 | 197 | 266 | 188 | 188 | 245 | 265 | 4 |
| **DRC 2009 B** | 115 | 197 | 272 | 188 | 188 | 245 | 0 | 1 |
| **DRC 2009 B** | 116 | 197 | 272 | 188 | 188 | 245 | 265 | 11 |
| **DRC 2009 B** | 117 | 197 | 272 | 188 | 188 | 251 | 265 | 4 |
| **DRC 2009 B** | 118 | 197 | 278 | 188 | 188 | 245 | 265 | 1 |
| **DRC 2013** | 119 | 197 | 284 | 188 | 181 | 221 | 0 | 1 |
| **DRC 2013** | 120 | 197 | 284 | 188 | 181 | 221 | 265 | 4 |
| **DRC 2009 A** | 125 | 203 | 249 | 188 | 188 | 245 | 265 | 1 |
| **DRC 2009 A** | 126 | 203 | 266 | 188 | 188 | 245 | 0 | 1 |
| **DRC 2009 A** | 127 | 203 | 266 | 188 | 188 | 245 | 265 | 13 |
| **DRC 2009 A** | 128 | 203 | 266 | 188 | 188 | 251 | 265 | 1 |
| **DRC 2011** | 129 | 203 | 272 | 188 | 188 | 245 | 265 | 1 |
| **Guinea 2012** | 130* | 209 | 278 | 197 | 202 | 0 | 0 | 1 |

The number of isolates corresponding to each MLVA type is indicated on the right. The environmental isolates are indicated with an asterisk.
