# Supplementary material for: Relationship between Distinct African Cholera Epidemics Revealed via MLVA Haplotyping of 337 Vibrio cholerae Isolates
Source: PLoS Negl Trop Dis. 2015 Jun 25;9(6):e0003817. doi: 10.1371/journal.pntd.0003817 (PMC4482140; doi:10.1371/journal.pntd.0003817)
Supplement: S1 Text — (DOCX) [file pntd.0003817.s002.docx]

### Main findings of the field investigation of cholera performed in Togo in December 2014

Togo has experienced cholera epidemics every year since 2000. However, the country has displayed a significant reduction in suspected cholera cases since the early 2000s. During the seven-year period from 2000 to 2006, a total of 7234 suspected cases were reported. Meanwhile, during the following seven-year period, only 983 cases were report in the country, yielding an average of approximately 140 cases each year. As of week 48 of 2014, a total of 281 suspected cases were reported in Togo.

To understand the dynamics of cholera in the country we performed a detailed assessment of the 2014 epidemic. We found that an initial epidemic struck the Lacs district during weeks 6-8 (indicated in dark blue in **SI** **Figure 1**), during which three deaths were reported. Of note, the first cases reported at the same period in Lomé during week 6 proved to be negative for *V. cholerae*. During week 9 and 11, one lab-confirmed case was reported in the districts of Golfe and Lomé, respectively, although these cases failed to give rise to epidemic expansion. Five suspected cases and two-cholera related deaths were again reported in Lacs during weeks 14-16.

The epidemic did not explode in Lomé until later in the year following a confirmed case on week 30. The epidemic peaked in the country on week 38. During this epidemic, a few suspected cases were also reported in Zio (1 case), Kloto (26 cases), Ave (9 cases) and Agou (6 cases). The epidemic then gradually subsided until week 45, when a second peak of cases occurred in Golfe during weeks 46-47 (**SI** **Figure 1**).

**SI Figure 1. Evolution of the 2014 cholera epidemic in Togo.** Each district reporting suspected cases is indicated in different colors.

In 2014, 55.8% of suspected cases were reported in Lomé. Furthermore, cases in Lomé were often residents of district D2 (59.9% of Lomé cases) or D3 (25.4% of Lomé cases) **(SI** **Figure 2**). We found that cases in Lomé were often associated with areas linked with fishing activity as well as flood zones, especially in Adakpamè, Bè Kpota, Anfamé, and Akodéssewa. In D2, cases appear to be associated with movement from Ghana and the large market close to the port.

**SI Figure 2. Evolution of the 2014 cholera epidemic in Lomé.** Each district within Lomé reporting suspected cases is indicated in different colors.

Many of the cases in D3 were reported in Katanga. This site is primarily a fishing community with people of various ethnic origins. There is significant movement between Katanga and Ghana. Traditional wells are the primary source of water. The water is slightly brackish and the level varies with the tides. The wells (1 well per 5-10 households) provide access to a significant amount of water for domestic use. The exact number of fishermen affected by the disease is unknown, as this population is very mobile and may evade disease surveillance. For example, fishermen from Ghana that contract cholera in Lomé sometimes prefer to return to Ghana for treatment.

In Lacs, both epidemics from 2013 and 2014 were associated with people traveling from abroad (e.g., Nigeria and Benin) for large annual traditional animist ceremonies, which usually occur during the dry season. For 2014, the first cases where people who attended the ceremony in Séko and then a few secondary cases occurred before the outbreak came to a halt. In Séko, the traditional animist ceremony was described to be rather “masculine”, which likely explains why males were more affected (**SI Figure 3**). Furthermore, the attendants of such ceremonies tend to be older, which correlates with the older average age of cases in Lacs (37.5 years), compared with Golfe (26.1 years), Kloto (31.9 years), D2 (27.6 years) and D3 (31.6 years). During the ceremony of 2014, it was stated that open defecation occurred adjacent to the site where animals were slaughtered for the meals. Activities also took place along a riverbank where attendants drink untreated water directly from the river, which could be easily contaminated by the open defecation practice.

**SI Figure 3. Cholera epidemic of 2014 in Togo: sex distribution by district.**

An unusual two-week peak of 27 cases was reported in Golfe during week 46-47. The major of the cases (67%; 18 of 27 cases) were reported from Agoè zongo, in which many of the initial cases were a group of children aged and two teachers all living in close proximity. All of the six cases who were interrogated, except for a 8-year-old boy who died on Nov 15^th^ (we spoke with his mother), obtain drinking water from the same borehole water station, although it is in very good condition.

The index case in Golfe during week 46-47 was likely a 17-year-old boy who first developed symptoms on Nov 13 at 2 AM. He stated that he had consumed water from the borehole water station and beans and cassava flour at home. He declared no history of travel or receiving visitors. Nobody in his immediate courtyard contracted the disease.

**Funding**

This consultancy was established via a partnership between UNICEF-Senegal and Aix-Marseille University, where the research team of Prof Renaud Piarroux is based. The funding covered the mission fees of Sandy Moore and Renaud Piarroux as well as fees associated with cartography and epidemiological assessments, genetic analyses and redaction of the final report.
